# Water isotope–temperature relationship variability across Antarctica set by atmospheric circulation

**DOI:** 10.1038/s41561-026-01961-y

**Published:** 2026-04-13

**Authors:** Mathieu Casado, Adriana Bailey, Christophe Leroy-Dos Santos, Elise Fourré, Vincent Favier, Cécile Agosta, Niels Dutrievoz, Christoph Kittel, Laurent Arnaud, Frédéric Prié, Pete D. Akers, Alexandre Cauquoin, Martin Werner, Leoni Janssen, Barbara Stenni, Giuliano Dreossi, Andrea Spolaor, Agnese Petteni, Joel Savarino, Amaelle Landais

**Affiliations:** 1 Laboratoire des Sciences du Climat et de l’Environnement (LSCE), CEA, CNRS, UVSQ, Université Paris-Saclay, IPSL, Gif-sur-Yvette, France; 2 Climate and Space Sciences and Engineering, University of Michigan, Ann Arbor, MI USA; 3 Institut des Geosciences de l’Environnement (IGE), Univ. Grenoble Alpes, CNRS, IRD, Grenoble INP, INRAE, IGE, Grenoble, France; 4 Department of Geography, UR SPHERES, University of Liège, Liège, Belgium; 5 Physical Geography Research Group, Department of Geography, Vrije Universiteit Brussel, Brussels, Belgium; 6 Discipline of Geography, School of Natural Sciences, Trinity College Dublin, Dublin, Ireland; 7 Institute of Industrial Science, The University of Tokyo, Kashiwa, Japan; 8 Alfred Wegener Institute Helmholtz Centre for Polar and Marine Research (AWI), Bremerhaven, Germany; 9 Department of Environmental Sciences, Informatics and Statistics, Ca’ Foscari University of Venice, Venice, Italy; 10 Institute of Polar Sciences – National Research Council of Italy (ISP-CNR), Venice, Italy

**Keywords:** Palaeoclimate, Cryospheric science

## Abstract

Water isotopes serve as tracers of hydrological processes and as proxies for past climates archived in ice cores. The isotopic signal is acquired throughout the hydrological cycle—through evaporation over the oceans, precipitation, which occurs as moisture is transported from lower to higher latitudes, and during post-depositional processes in which isotopic exchange between snow and atmospheric moisture occurs. Owing to these multiple influences, the relationship between isotope ratios in ice and local temperature varies across Antarctica, and distinct relationships are found when evaluating isotope ratios and temperature across space (for example, in surface snow) compared with temporal correlations at the same site (for example, in precipitation). Here we report measurements of water vapour isotopic compositions from a traverse across East Antarctica, as well as at two fixed sites: the coastal station Dumont D’Urville and Dome C on the plateau. Combining snow and vapour isotopic data, we demonstrate that the temporal and spatial isotope–temperature relationships are distinct because of differences in how the rainout fraction varies across time and space. Our findings support a shift from thinking about the isotope–temperature relationship in terms of distinct temporal and spatial slopes to recognizing that the relationship varies along a continuum based on known dependencies between circulation dynamics and mean climate state. By distilling moisture along moist isentropic transport paths, we can predict the isotope–temperature relationship across either time or space using a physical understanding of large-scale moisture transport under different climatic conditions.

## Abstract (web summary)

Atmospheric circulation patterns and mean climate conditions lead to heterogeneity in water isotope–temperature relationships across Antarctica, and accounting for these effects allows for more robust interpretation of temperature proxy records from ice cores.

## Main

The empirically observed relationship between δ^18^O and local temperature forms the basis for isotopic palaeothermometry^1^—where linear regressions are used to develop transfer functions that allow us to infer past temperatures from ice core records. Owing to the lack of centennial (or longer) surface air temperature records in Antarctica, the palaeothermometer has traditionally been derived from spatial variations in 10-m snow temperatures (proxies for mean annual temperature) and the isotopic composition of multi-metre snow samples, which typically integrate several decades of accumulation^2,3^. However, both modelling and observations indicate that the isotope–temperature relationship varies in space^4^ and time^5^. For example, isotopic measurements of surface snow yield spatial isotope–temperature gradients of about 0.8–1.2‰ °C^−1^ (refs. ^4,6^), while precipitation samples, mostly used to study temporal relationships, suggest slopes of about 0.3–0.6 °C^−1^ (refs. ^7–10^). Even distinct temporal relationships have been found depending on the timescale^5^. Consequently, a single linear relationship is insufficient for reliably reconstructing past temperature from ice cores.

It is well documented that several factors can influence the final isotope ratio record in ice, including (1) oceanic evaporative conditions^11^, transport pathways^12^, precipitation seasonality^13^ and intermittency^14–16^; and (2) post-depositional processes^17–19^, including exchanges between the atmosphere and surface snow that can modify isotopic signatures^20–22^. However, a critical factor linking the water isotopic composition of glacial ice to local air temperature is the meridional temperature gradient, which increases as polar temperatures decrease in response to large-scale climate variability^23^. A stronger gradient causes a greater fraction of moisture to condense and precipitate as the air moves poleward; and because heavy isotopes are more effectively removed during precipitation than light isotopes, this lowers the isotope ratio of the moisture arriving at Antarctica^24^. These dependencies suggest that a physically based transfer function—one that links Antarctic isotope ratios to underlying circulation dynamics—would be a desirable additional constraint on using ice isotope ratios to infer past temperature.

Rayleigh distillation is a modelling framework commonly used to predict the evolution of the isotopic composition of the atmosphere as moisture is transported away from its source. It models the isotope ratio based on the initial isotopic composition of the moisture source, the fractional removal of moisture by precipitation and the temperature at which condensation occurs. Although it has been applied to predict isotope ratios over Antarctica^25^, it is typically unsatisfactory when applied in a one-dimensional sense (that is, as a function of latitude only) because it ignores the effects of atmospheric mixing. Two-dimensional modelling approaches suggest that Antarctic isotope ratios are better reproduced when both distillation and mixing processes are taken into account^23,26^. In comparison, more recent results suggest that polar isotope ratios can be adequately simulated by a Rayleigh distillation as long as the distillation is modelled along surfaces of constant moist entropy^27^, along which mixing may be considered negligible.

To elucidate the physical basis for why different temporal and spatial isotope–temperature relationships emerge, here we test whether we can replicate the variable isotope–temperature slopes derived in precipitation and surface snow. We do so using an extensive suite of water vapour measurements from Antarctica and by leveraging the idea that poleward moisture transport pathways are well modelled by moist isentropic surfaces^27^. The measurements allow us to take what has traditionally been an empirically derived relationship and show that the dependence of the moist circulation on climate is what ultimately determines the isotope–temperature slope, and how it varies between warmer and colder states. Unlike precipitation or surface snow, which are easier to sample and have been more commonly used to study the isotope–temperature relationship, water vapour isotopic composition is a direct tracer of distillation processes in the hydrological cycle, unaffected by precipitation intermittency or post-depositional effects. Our results move the isotopic palaeothermometry discussion from a site-by-site specific treatment of the isotope–temperature relationship to one based on a physical understanding of generalizable isotopic dependencies on moisture transport under different hydroclimate conditions.

## Isotopic time series

**Fig. 1:**
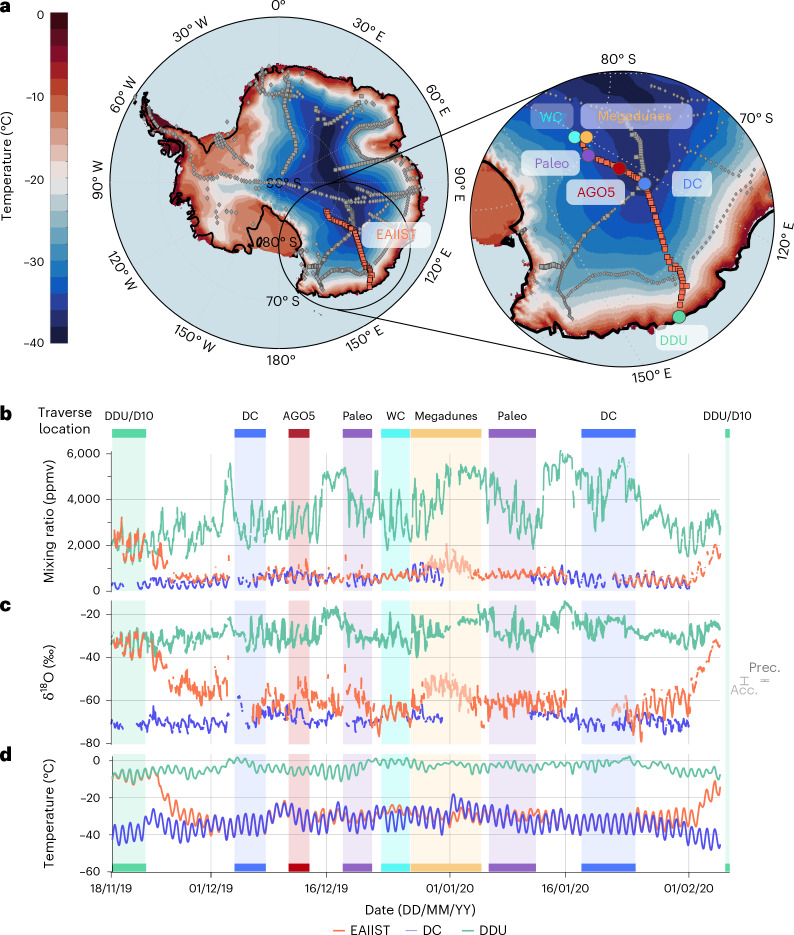
EAIIST traverse map and isotopic time series. **a**, Route (orange squares) and previous traverses sampling snow isotopes in East Antarctica (grey diamonds^4^ and squares^6^) over mean Modèle Atmosphérique Régional (MAR) model temperatures (between November 2019 and February 2020). Inset shows stop sites: DDU, DC and four ice core sites (AGO5, Paleo, WC and Megadunes). **b**–**d**, Mixing ratio (**b**), δ^18^O (**c**) and MAR-estimated air temperature (**d**) from 16 November 2019 to 3 February 2020 for EAIIST (orange), DDU (green, including D10, which is 10 km inland from DDU) and DC (blue). Coloured bars show the duration of traverse stops (colours match those in **a**). EAIIST data are shown with reduced opacity for Megadune and DC stops due to possible vapour measurement contamination (Methods). Light (Acc.) and dark (Prec.) bars show measurement accuracy and precision, respectively. Maps in **a** generated with MATLAB.

Advances in field-deployable infrared spectrometers have enabled in situ measurements of water vapour isotopic composition^28–33^. Here we present parallel measurements of water vapour isotopic composition from East Antarctica, including the coastal Dumont D’Urville (DDU) station, the inland Dome C (DC; 3,233 m above sea level) and the East Antarctic International Ice Sheet Traverse (EAIIST) from DDU to 80° S via DC and back^34^ (November 2019 to February 2020; Fig. 1a). Because atmospheric moisture forms precipitation, vapour and precipitation isotopic compositions should evolve in parallel under the influence of Rayleigh distillation^25^. Thus we use vapour data to assess local and remote controls on vapour and snow isotopic composition, and to quantify differences between spatial and temporal (seasonal to interannual) isotope–temperature relationships that would affect interpretation of ice core records.

**Extended Data Fig. 1:**
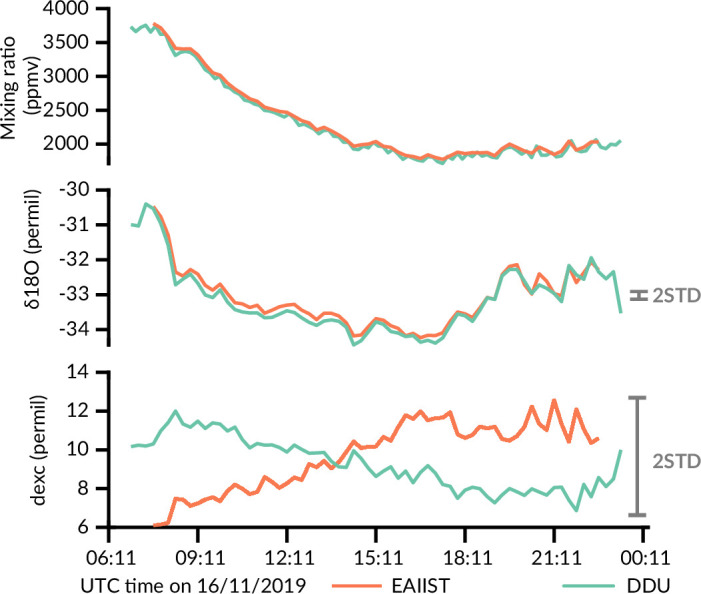
Evaluation of accuracy of multiple instrument measurements. Comparison of measured mixing ratio, δ^18^O, and d-excess for both instruments on the same inlet over 14 hours.

Figure 1 presents three time series of water vapour isotopic composition obtained between 18 November 2019 and 4 February 2020 using three infrared spectrometers: one at DDU, one at DC and one mobile instrument mounted in a sled-borne laboratory on the EAIIST traverse. The large amplitude of the δ^18^O signal—about 40‰ spatially and 10‰ temporally—greatly exceeds the measurement precision and accuracy (0.2‰ and 1.5‰, respectively; Extended Data Fig. 1 and Methods). The observations cover the full range of moisture conditions expected during summer in East Antarctica, with mixing ratios from 50 to 6,000 parts per million by volume (ppmv).

At DDU, the mixing ratio shows a clear diurnal cycle (~1,500 ppmv peak-to-peak), modulated by synoptic events that increase humidity to 6,000 ppmv^32^. In contrast, isotopic variability at DC is dominated by diurnal cycles in the boundary layer, driven by variable resupply of near-surface moisture by sublimation^17,29,35^. The EAIIST traverse covered a mixing ratio range between those observed at DDU and DC, behaving similarly to DDU until the route reached the plateau on 24 November, when humidity dropped to values typical of DC, nearly 1,000 km away. On the return leg (after 1 February), an equally abrupt humidity increase marked the descent back towards the coast (Fig. 1).

The EAIIST traverse vapour δ^18^O similarly spans the values measured at DDU and DC (Fig. 1c). As EAIIST moved inland, δ^18^O transitioned progressively between DDU and DC values, with intermittent diurnal cycles superimposed. Back-trajectory analysis suggests that at the day-to-day scale, air masses reaching DDU, DC and EAIIST are primarily shaped by large-scale advective influence, not local boundary layer contributions (Supplementary Section 2). Moreover, in comparing vapour and surface snow, we find that while diurnal isotopic variability at DC (purely temporal) and along EAIIST (spatio–temporal) is largely influenced by local snow–atmosphere exchange (Supplementary Section 2), the day-to-day vapour variations over the plateau are influenced by large-scale moisture transport (Supplementary Section 3).

## Spatial and temporal isotope–temperature relationships

Using our dataset, we evaluate the temporal isotope–temperature relationships at DC and DDU and the spatial relationship from EAIIST, which is not purely instantaneous but nevertheless dominated by spatial variability (Table 1). At DC, the vapour-derived temporal slope (0.35‰ °C^−1^ for summer (November–February (NDJF); *P* < 0.05)^29^ matches previously published estimates from precipitation (0.37‰ °C^−1^ NDJF; 0.48‰ °C^−1^ seasonal cycle; *P* < 0.05)^9^. Similarly, the vapour-derived spatial slope from EAIIST (1.05‰ °C^−1^; *P* < 0.05) aligns with the surface snow near DC (1.0‰ °C^−1^; *P* < 0.05)^4^ and precipitation between DC and DDU (0.95‰ °C^−1^, two-end-member estimate calculated by dividing the difference of mean isotopic composition and temperature between DC and DDU). This coherence among vapour, precipitation and snow strongly suggests that the difference between spatial and temporal isotope–temperature slopes reflects the dominant influence of large-scale atmospheric dynamics, and the associated distillation processes, rather than local microphysics.

**Table 1:** Summary of observed isotope–temperature relationships and modelling outputs

	Type of proxy	Spatial relationship (‰ °C^−1^)	Temporal relationship DC (‰ °C^−1^)		Temporal relationship DDU (‰ °C^−1^)	
			NDJF	Seasonal	Interannual	Seasonal
Observations	Vapour	1.05*	0.35* (ref. ^29^)	-	-	0.52* (ref. ^33^)
	Surface snow	1.0* (ref. ^4^)	-	0.49^a^ (ref. ^17^)	-	-
	Precipitation	0.95^a^	0.37* (ref. ^9^)	0.48* (ref. ^9^)	0.52* (ref. ^9^)	0.4* (ref. ^33^)
	Ice cores	-	-	-	0.5* (ref. ^52^)	-
Modelling	iCAM5 precipitation	1.5*	0.4*	0.5*	0.2	0.2*
	Isentropes iCAM5	1.3*	-	0.6^a^	-	0.3*
	ECHAM6-wiso precipitation	1.8*	0.8*	0.6*	0.9*	0.4*
	Isentropes ECHAM6-wiso	1.3*	-	0.5^a^	-	0.4*

Modelling outputs are derived using the nearest GCM gridpoint and isentropic framework. Significant linear regressions are indicated *P < 0.05. Slopes with reference numbers in parentheses are from the literature. Spatial vapour relationships are for EAIIST, which include some temporal variations as well. The surface snow spatial relationship is only inferred, using data from the Indian sector of East Antarctica.

^a^Result obtained using end-member difference calculations (as opposed to linear regression).

## A physical basis for different spatial and temporal isotope–temperature relationships

**Fig. 2:**
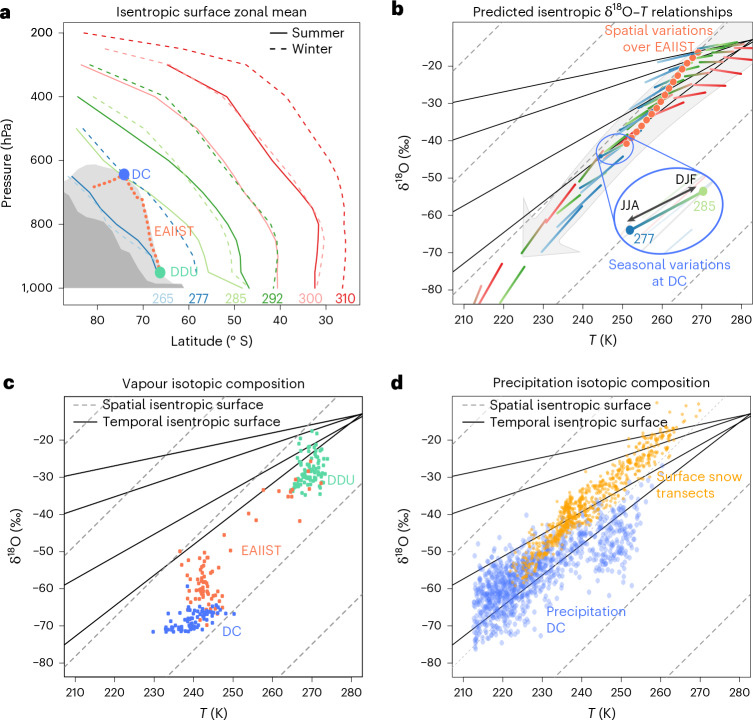
Spatio–temporal isotope data and isentropic contours. **a**, Isentropic surfaces (*θ*_s_) versus latitude and pressure in the Southern Hemisphere (coloured lines) following ref. ^27^. The DDU, DC and EAIIST route is marked against EAIIST’s latitude–altitude profile (light grey) and the zonal-mean Antarctic profile (dark grey). **b**, Predicted isotope–temperature (*T*) variation: DC seasonal cycles shown by shifts from one *θ*_s_ surface to another; EAIIST spatial gradient (orange dotted line) crosses moist isentropes. The grey arrow can be interpreted in two different ways: under constant hydroclimate conditions, it can represent the increased rainout fraction spatially, whereas for changing hydroclimate conditions, it represents how the system becomes colder and drier. Colours match *θ*_s_ contours in **a**. **c**, Vapour δ^18^O versus temperature at DDU (green), DC (blue) and EAIIST (orange). **d**, Comparison with snow transect and DC precipitation cycles. Grey dashed lines show 1‰ °C^−1^ slope with different zero crossing values; solid black lines show slopes of 0.2, 0.4, 0.6 and 0.8‰ °C^−1^ (from top to bottom).

**Extended Data Fig. 2:**
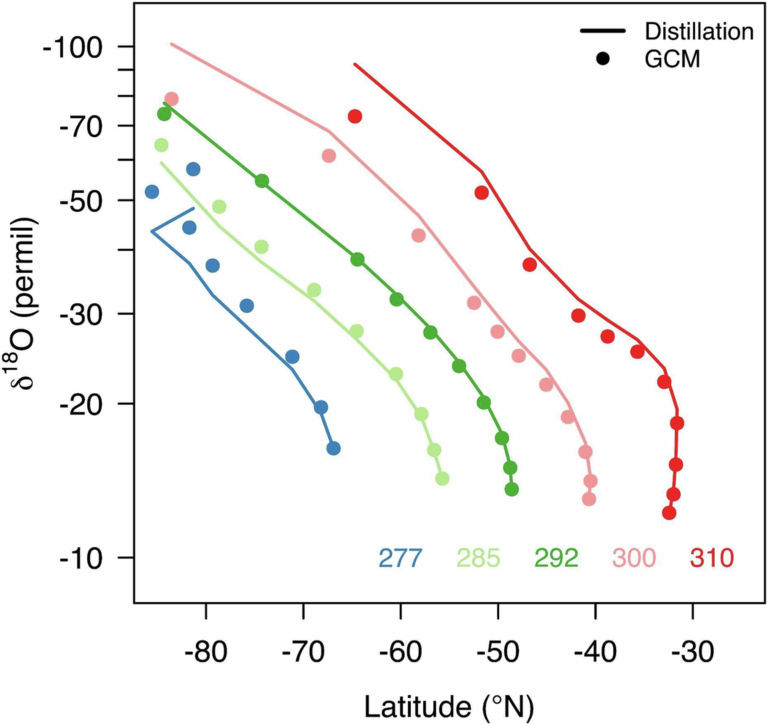
Isentropic surfaces in both modelling frameworks. A comparison of water vapour isotope ratios averaged across the moist isentropic moisture transport paths in iCAM5 (filled circles) and modelled by applying Rayleigh distillation to the temperature and mixing ratio values of the moist isentropic surfaces (${{\rm{\theta }}}_{{\rm{S}}}$ labelled lines, units K).

Previous studies have argued that the reason temporal and spatial isotope–temperature relationships differ is because distinct locations are influenced by different moisture source regions and transport pathways^4^. General circulation models (GCMs) are able to reproduce this difference^36^, although their complexity often obscures clear attribution. Here we present a simple theoretical framework that accounts for these differences and is generalizable across timescales and sites. Starting from the premise that poleward moisture transport largely conserves moist entropy in the zonal mean^27^, we use moist isentropes (examples shown in Fig. 2a and Extended Data Fig. 2) to identify zonal-mean moisture transport pathways^27^ and extract the temperature and water vapour isotopic evolution along these quasi-Lagrangian transport paths. The isotope–temperature relationship for a given Antarctic site is obtained from the temperature and isotope ratio values from along the moist isentrope that intersects the site’s latitude and elevation (Methods).

We define isentropes using the moist entropy potential temperature (*θ*_s_). Temperature and mixing ratios used to calculate *θ*_s_ are derived from simulations with two isotope-enabled GCMs (isoGCMs): the Community Atmospheric Model version 5 (iCAM5^37^) and ECHAM6-wiso^38^. We select isentropes that pass through DDU and DC and provide the widest possible seasonal overlap. We define the moisture sources for each site based on where the isentropes intersect the Earth’s surface (near 1,000 hPa) and take the simulated near-surface isotope values from each GCM at these intersection points to be the isotope ratios of the source.

Using the zonal δ^18^O and temperature values from the *θ*_s_ surfaces that intersect DDU and DC (shown as individual lines with colour transitions between austral summer and winter in Fig. 2b), we derive a moist isentropic prediction for the isotope–temperature relationship. We find that temporal slopes at DC and DDU are about half the predicted spatial slope between the sites in NDJF, consistent with both vapour and precipitation observations (Table 1). For example, using input from iCAM5 to generate the moist isentropic surfaces, we obtain a seasonal change in *θ*_s_ from 277 K to 285 K (inset in Fig. 2b), implying a slope of about 0.6‰ °C^−1^ at DC. In comparison, the observed temporal isotope–temperature relationship between isotopic composition and temperature is about 0.5‰ °C^−1^ at seasonal and interannual timescales^9^.

Furthermore, despite the simplicity of the approach, slope estimates from the moist isentropic transport pathways are qualitatively similar to those derived from the nearest GCM gridpoint outputs (Fig. 2b). This suggests that the isotope–temperature relationship in isoGCMs is mainly controlled by large-scale moisture transport rather than local site-specific processes^23,38,39^. We report only a reduced number of significant digits in Table 1 due to the large spatial variability in GCM gridpoint estimates^40^ (Supplementary Section 4).

Our results help to clarify why temporal and spatial slopes differ. By tracing the moist isentropic surfaces at DC and DDU back to their source regions, we see that these two sites lie at very different distances from their respective moisture sources. As a result, the moisture arriving at DC undergoes a greater degree of distillation and is more isotopically depleted than moisture arriving at DDU. The difference in fractional moisture removal by distillation (Δ*f*; Methods) between DC and DDU is 0.84 during austral summer, while the seasonal difference at DC is only 0.02. Although the *θ*_s_ value of DC’s moisture transport path changes seasonally by 8 K, its geometry remains nearly unchanged (Fig. 2a). Consequently, seasonal differences in fractional rainout are governed mainly by nonlinear variations in moisture content with temperature (consistent with Clausius–Clapeyron scaling), which are very small given the low temperature and humidity values that typify the plateau. The end result is that the isotope ratio’s sensitivity to temperature is much larger in space than in time.

Additionally, our results help to clarify why temporal slopes can vary depending on the climate state (or similarly, depending on the time period of the analysis). As shown in Fig. 2b, the seasonal slope changes both with location (distance from moisture source, indicated by the grey arrow) and with surface thermodynamic conditions. Consequently, there is no single temporal isotope–temperature relationship, but rather a range of relationships that can exist for a given set of isotopic and thermal conditions.

## Consequences for interpreting ice core records

**Fig. 3:**
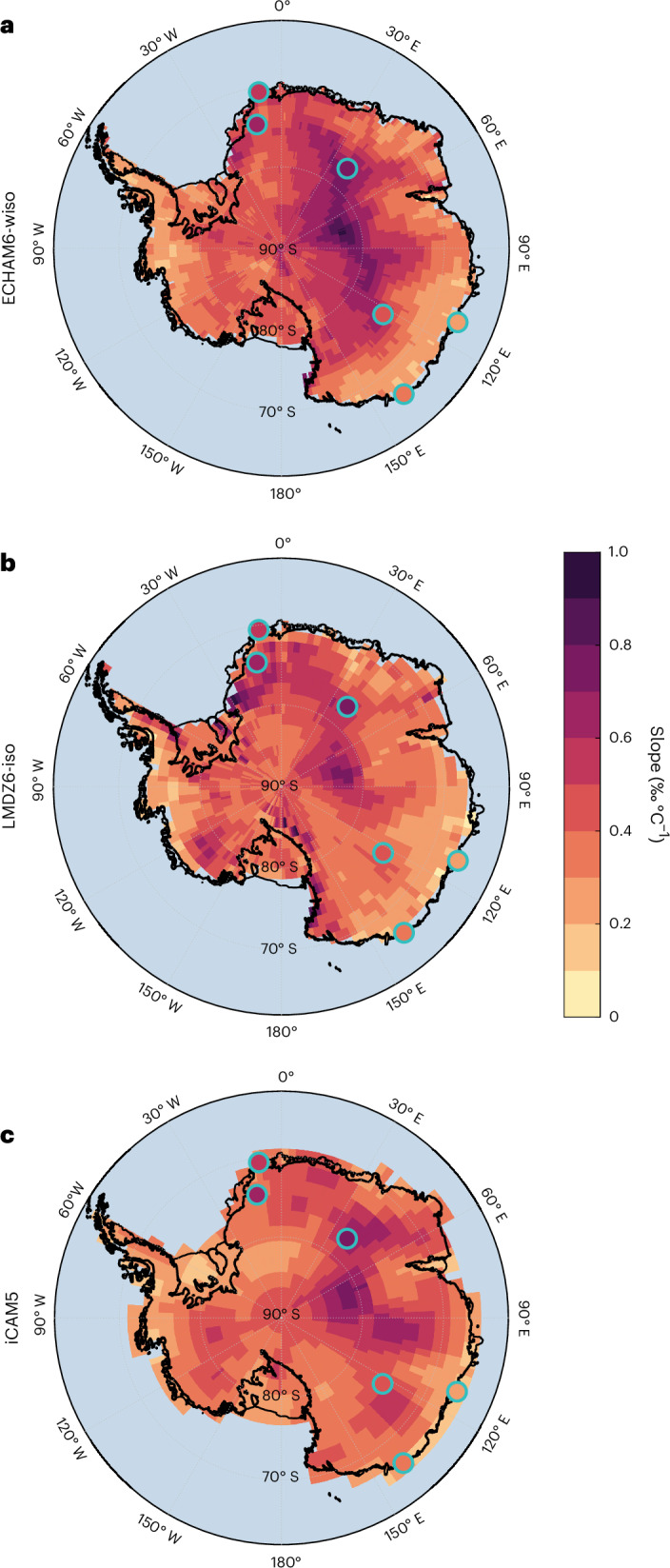
Modelled isotope–temperature relationships in Antarctica. **a**–**c**, Isotope–temperature slope variability across Antarctica using precipitation isotopic composition and 2-m-temperature (T2m) model outputs from ECHAM6-wiso (**a**), LMDZ6-iso (**b**) and iCAM5 (**c**). Literature values for given locations are indicated with teal-coloured circles. All datapoints exhibit a statistically significant linear relationship (one sided, *P* < 0.05). Map data from Bedmap2.

Isotope-enabled GCMs incorporate many factors that affect the predicted isotope–temperature relationships at ice core sites, but parameter uncertainties propagate through predictions. In comparison, computing the Rayleigh distillation along moist isentropic surfaces requires only information about the zonal-mean thermodynamic state, which is typically resolved and predicted with higher confidence. This simple framework provides a physical basis for understanding complex predictions of isoGCMs, which allow us to evaluate confidence in the isotopic palaeothermometer at specific ice core sites, for different climate conditions, time periods and timescales. In Fig. 3, we present the spatial patterns of the seasonal isotope–temperature relationships predicted by ECHAM6-wiso, LMDZ6-iso and iCAM5 compared with values found in the literature. The similarity between the spatial patterns of isotope–temperature relationships from the three isoGCMs and the spatial patterns of moisture source regions and equivalent potential temperature at the surface (a close proxy for moist entropy potential temperature)^27^ suggests that source latitude and characteristic distillation pathway are the dominant factors influencing spatial variability in the isotope–temperature relationship—factors that are captured by the moist isentropic framework.

Although our approach provides a physical basis for explaining variations in the isotope–temperature relationship in either vapour or precipitation, it is important to note that climate reconstruction must also consider that the isotopic signal in ice cores includes noise from precipitation intermittency^15^, stratigraphic mixing^41^ and post-depositional processes^19–22^. This is crucial because water isotopes record only days with precipitation, so the mean temperature of precipitation days may not match the mean annual temperature^42^. For DC, for example, the correlation between mean annual temperature and mean temperature of precipitation days in a year is only *r* = 0.27 (*P* > 0.1). Additionally, although our results for DC cannot be directly transferred to other sites, the approach can be generalized: by demonstrating that the same relationship is visible in vapour (daily) and precipitation (random sub-set of days), we suggest that the isotope–temperature relationship is attributed to the large-scale hydrological cycle and should not be fundamentally biased by precipitation intermittency. As data collection efforts in Antarctica expand, application of this framework for individual moist intrusions whose precipitation impacts are especially large^43,44^ could help investigate the impact of extreme events, at the tail end of the distribution, to the isotopic signal.

Our results confirm that the spatial isotope–temperature relationship is different from temporal slopes at the seasonal scale, and temporal slopes should not be assumed constant at longer timescales. At centennial to millennial scales, reconstructions that combine multiple palaeothermometers often yield larger isotope–temperature slopes for the last deglaciation (for example, 0.7–1.2‰ °C^−1^ at DC)^36,45^. Because meridional temperature gradients probably shift on such timescales, the distillation pathway can also change, modifying the isentropic surface associated with a given ice core site and widening the *θ*_s_ range beyond that seen for present-day seasonal variability. For instance, during warmer periods moisture may originate farther from the polar regions than during the last glacial period^46^, placing DC on a colder isentropic surface and increasing the δ^18^O–temperature slope for multi-millennial scales^36,45^. The rainout fraction could also vary substantially with changes in ice sheet topography^47^ or sea ice extent^48^. To consider distillation along the moist isentropic surfaces at these longer timescales, model simulations capable of representing ice sheet and atmospheric circulation changes under different climate states are required. However, relying strictly on GCMs to provide the isotope–temperature relationship for interpreting ice core records will lead to the reconstructions being model-dependent, which would not be suitable for tuning GCMs at long timescales, for instance. This suggests that independent calibrations from other temperature proxies might be more favourable. Alternative proxies such as inert gases or borehole thermometry can help to constrain past temperature estimates. For example, borehole measurements have been used in Antarctica to quantify warming over recent decades^49^ or the last deglaciation^45^, but are limited at other timescales due to thermal diffusion smoothing the signal. Inert gases can provide a complementary temperature reconstruction in Antarctica^50^ but may also be affected by firn air transport processes^51^. Water isotopes remain essential for temperature reconstructions from deep ice cores at all timescales.

In summary, the distillation of moisture along moist isentropic surfaces offers a physically based explanation for the observed differences between spatial and temporal isotope–temperature slopes. In this framework, spatial variations mainly reflect changes in fractional removal of moisture by precipitation along transport paths, while temporal variations are largely temperature-dependent. By comparing vapour isotopic variations temporally and spatially across Antarctica with this theoretical framework, we demonstrate that the difference in slopes is the result of changes in large-scale moisture transport. Our findings have important implications for the interpretation of ice core isotope records, as they move us away from the traditional isotopic palaeothermometer defined by a linear relationship between isotope and temperature variations, towards thinking about the isotope–temperature relationship as a continuum that depends on the dominant hydroclimate conditions. Our framework provides an intuitive way to understand and evaluate predictions of Antarctic isotope–temperature covariability in more complex GCMs.

## Methods

### The EAIIST traverse

The EAIIST is a French, Italian and Australian collaboration aimed at studying some of the driest areas of the East Antarctic Plateau. The traverse covered largely unexplored regions between DC and the South Pole (Fig. 1), providing an opportunity to study in detail a megadune area^53^ where accumulation rates are thought to be an analogue of glacial conditions at DC and other deep ice core drilling sites^54^.

The traverse began on 23 November 2019 when it departed from DDU station (66° 40′ S, 140° 00′ E). It passed through Concordia station (DC; 75° 06′ S, 123° 20′ E) from 3 to 7 December 2019, reached the Megadunes area on 26 December, returned to DC on 18 January 2020 and arrived back at DDU by 4 February 2020. In total, the caravan covered approximately 3,500 km. Besides preparation periods at DDU and DC, the traverse stopped at Paleo from 18 to 21 December (79° 45′ S, 125° 46′ E), Wind Crust (WC) from 23 to 26 December (80° 47′ S, 122° 10′ E), Megadune from 26 December to 4 January (80° 34′ S, 121° 47′ E) and again at Paleo from 5 to 11 January.

The caravan, pulled by five Caterpillar tractors and one Kassbohrer to prepare the track, included two science laboratories (white containers in Supplementary Fig. 7a): a warm lab for atmospheric measurements (for example, water vapour isotopes and dust) and in situ continuous flow analysis of surface snow cores; and a cold lab for storing and cutting ice cores. Two living containers (orange containers in Supplementary Fig. 7a) accommodated the crew.

In addition to routine geophysical and geochemical measurements at DDU and DC, 18 ice cores were drilled during the traverse at AGO5, Paleo, WC and Megadunes. These cores will help to assess the spatial variability of palaeoclimate proxies archived in ice. Automatic weather stations were installed at Paleo and Megadunes to evaluate how representative DC is compared with the broader East Antarctic Plateau.

### Water vapour isotopic composition monitoring

Water vapour isotopic composition was measured using a Picarro L2140-i installed in the caravan’s warm lab. A 5-m inlet drew air in from the roof of the lab, 5.4 m above the snow surface, via a dedicated membrane pump. The warm lab was positioned near the front of the convoy (second white container in Supplementary Fig. 6a) to minimize contamination from tractor and generator exhaust. The convoy was generally arranged so that prevailing winds blew emissions away from the inlet, especially during extended stops. A close-up of the warm lab (CLIMCOR, Supplementary Fig. 6b) shows the railing that supported the inlet. As the upper metre is hollow, snow accumulating on the roof should have minimal interaction with the inlet.

The Picarro analyser was strapped tightly to a desk inside the warm lab (Supplementary Fig. 6c) to limit movement during travel over rough terrain. High-density foam placed above and below the instrument reduced vibration. Despite some extremely rough segments, the instrument ran continuously throughout the traverse.

In addition, data from two Picarro isotope analysers at DDU and DC were included. At DDU, the analyser was installed in the Glacio shelter on Petrel Island, facing the continent. An initial test ran for 2 months in 2016–2017^32^, followed by a permanent setup in 2018^33^. At DC, the analyser operates in the Snow shelter, 1 km upwind of the station. After two summer test seasons in 2014–2015 and 2015–2016^29^, a permanent instrument was installed in 2018^55^, although performance limitations under extremely dry winter conditions prevented year-round measurements^56^.

### Calibration and data curation

Water mixing ratio and isotopic composition (δ^18^O, δD) were measured nearly continuously during the EAIIST traverse. Although calibration was not possible along the route, two calibration exercises were conducted: one at DDU before departure and one at Laboratoire des Sciences du Climat et de l’Environnement (LSCE) 5 months after return, following the protocol in ref. ^57^. First, the analyser’s water mixing ratio was compared with true values from weather stations (at DDU) or a dew point generator (at LSCE; Supplementary Fig. 7). Next, the humidity dependence of isotopic composition was assessed by injecting a standard (NEEM, δD = –257.2‰; δ^18^O = −33.5‰). Finally, two standards (NEEM and FP5, δD = −395.9‰; δ^18^O = −50.64‰) were injected at mixing ratios of 1,500 and 1,000 ppmv to link measured δD and δ^18^O to true values. The consistent results from field and lab calibrations were used to correct the raw data.

Because the inlet was mounted on top of the vehicle, some contamination was still detected. Data were excluded when mixing ratios exceeded 3,000 ppmv or when 15-min variability was greater than 100 ppmv—conditions unlikely under natural Antarctic circumstances.

Vapour isotopic data from the traverse were compared with simultaneous records at DDU and DC. At these sites, the analysers are equipped with low-humidity vapour generators^57^. The δ^18^O and δD calibration steps described above are performed on site, with humidity effects checked annually and drift assessed every 48 hours by injecting a known standard.

The root mean square difference between the two calibrations (field and lab) provides a conservative estimate of absolute accuracy: ±1.5‰ for δ^18^O and ±3.9‰ for δD (2 × root mean square error, RMSE, by analogy with 2*σ* precision).

### Evaluation of instrument performance

To check precision under realistic conditions, the EAIIST instrument was temporarily co-located with the permanent DDU analyser and connected to the same inlet. After independent calibrations, both instruments produced closely matching humidity and δ^18^O measurements for 14 hours spanning the full summer humidity range at DDU (Extended Data Fig. 1). The precision, estimated as 2*σ* of their difference, was 0.22‰ for δ^18^O, in line with independent Arctic results^58^.

Given the amplitude of observed signals along the traverse (Fig. 1), these precision levels are sufficient to ensure the features analysed are genuine, not noise artefacts.

### Comparison with precipitation and surface snow isotopes

Daily precipitation samples were collected at DC^59^. Daily temperatures and isotopic composition were used to evaluate the δ^18^O–temperature relationship at multiple timescales: daily values for intra-seasonal and seasonal slopes, and annual means for interannual slopes. Precipitation samples are also available from DDU^33^.

Surface snow samples were collected at DC and along EAIIST. At DC, sampling followed the protocol detailed in ref. ^17^: snow was taken from ten locations within 100 m^2^ areas every 25 km to depths of 1.5 cm and mixed. Samples were georeferenced and analysed at LSCE (Paris, France) and University Ca’ Foscari (Venice, Italy) on Picarro instruments, and compared with the database in ref. ^4^.

### Moist isentropic surfaces

We define quasi-Largrangian moisture transport paths using zonal-mean moist isentropic surfaces, although our results would be almost indistinguishable using equivalent potential temperatures instead^60^. Although our method follows ref. ^27^, here we use the moist entropy potential temperature (*θ*_s_) as a proxy for moist entropy, and approximate its value (neglecting liquid/ice terms) using:

$${\theta }_{{\rm{s}}}\,=\theta \,(1+\lambda \times \,r)$$

where *θ* is the potential temperature, *λ* is the difference between water vapour and dry air reference entropies, normalized by dry air specific heat at constant pressure, and *r* is the water vapour mixing ratio^60^. Had we used equivalent potential temperature instead^27^, our analysis would have produced nearly identical results in the higher-latitude environment relevant to Antarctica.

For regular pressure levels, we average the latitudes, temperatures and mixing ratios for all points south of 25° S whose *θ*_s_ values fall within ±2.5 K of the six *θ*_s_ values specified a priori (265 K, 277 K, 285 K, 292 K, 300 K, 310 K; see Fig. 2). Zonal-mean calculations are performed separately for December–January–February (DJF) and June–July–August (JJA). Equivalent but mass-weighted isotopic averages provide the isotopic evolution along each moisture transport path. All variables used to define the moist isentropic surface and its isotopic evolution are derived from isoGCM simulations (either iCAM5 or ECHAM6-wiso; see next subsection).

From previous work^27^ and additional testing, we know that the isotopic evolution along the GCM moist isentropes closely matches a familiar Rayleigh distillation given by:

$${\rm{d}}{\rm{ln}}(R)=(\alpha -1)\,{\rm{d}}{\rm{ln}}(f)$$

where *R* is the isotopic ratio, *f* is the change in mixing ratio (*r*/*r*_0_), here considered along the moist isentropic surface, *α* is a temperature-dependent scaling factor^61^ and dln stands for the derivative of the logarithmic function. Each moist isentrope is characterized by a unique initial set of conditions (*r*_0_, *T*_0_ and ẟD_0_). As a result, the distillation function varies by surface. Extended Data Fig. 2 shows how zonal-mean isotope ratios in the Community Earth System Model mirror the ẟ^18^O values predicted by distilling moisture along each surface. For the purposes of simplifying our results here, we thus use the zonal-mean isotope ratios from each GCM to estimate the isotope–temperature slopes. Because the Rayleigh distillation can be calculated for each value of *θ*_s_, leading to different change in mixing ratio for a given temperature, this approach leads to a generalization of the Rayleigh distillation to two dimensions, where classical Rayleigh models are one-dimensional.

To determine the isotope–temperature relationship predicted by the moist isentropic transport pathways, we identify the *θ*_s_ surfaces that intersect DC and DDU in different seasons and take the GCM isotope ratio and temperature values at those points. The temporal slope is determined by differencing the isotope ratio and temperature values characteristic of different seasons at a single site. The spatial slope is determined by differencing the isotope ratio and temperature values between sites for a single season. Temporal slopes use DJF–JJA differences at each site; spatial slopes compare the sites’ DJF differences.

Fractional moisture removal by precipitation (1 − *f*) is defined by the proportional decrease in mixing ratio (*r*) along the moist isentropic path with respect to the mixing ratio of that transport pathway where it intersects Earth’s surface (*r*_0_):

$$1-f\,=\frac{{r}_{0}-r}{{r}_{0}}\,$$

Here, *f* is equivalent to the fraction of water vapour remaining used in a Rayleigh distillation calculation.

### GCM outputs

iCAM5 outputs come from an isotope-enabled simulation with CAM5 coupled to the isotope-enabled Community Land Model (CLM4) with prescribed sea surface temperatures, sea ice, greenhouse gases and aerosols for the years 1975–2014^62,63^. ECHAM6-wiso runs^64^ are nudged to the fifth generation ECMWF reanalysis (ERA5) temperature and vorticity^65^ for the years 1979–2021. LMDZ6-iso output comes from the isotope-enabled version of LMDZ6^66^ and is evaluated in Antarctica^67^. The simulation is nudged towards 6-hourly three-dimensional fields of temperature and wind from the ERA5 reanalysis using a 12-hour relaxation timescale, except below the sigma-pressure level corresponding to 850 hPa above sea level. We use the standard low-resolution horizontal grid of LMDZ6, with 144 × 142 points corresponding to a 2.0° longitude by 1.67° latitude resolution, and 79 vertical levels. Surface ocean boundary conditions are derived from ERA5 reanalysis monthly mean sea surface temperature and sea ice concentration fields. The simulation covers the years 1979–2022.

To compare the isotope–temperature relationships derived from the moist isentropic transport pathways with the nearest GCM gridpoint, we use the following geolocational coordinates in the isoGCMs. For DC, the nearest iCAM5 gridpoint is an average of 122.5 and 125.0 °E, 74.84° S and model level 30 (~651 hPa in DJF); the nearest ECHAM6-wiso gridpoint is 123.75° E, 75.27° S and model level 93 (two levels above the surface to avoid local fractionation artefacts); for DDU, the nearest iCAM5 gridpoint is 140° E, 65.37° S and model level 30 (~954 hPa in DJF); the nearest ECHAM6-wiso gridpoint is 139.69° E, 65.92° S and model level 93 (again two levels above the surface to avoid local fractionation artefacts).

## Online content

Any methods, additional references, Nature Portfolio reporting summaries, source data, extended data, supplementary information, acknowledgements, peer review information; details of author contributions and competing interests; and statements of data and code availability are available at 10.1038/s41561-026-01961-y.

## Supplementary information

Supplementary Information Supplementary Text 1–6, Figs. 1–8 and references 1–29.

## Data Availability

Water isotopic composition data measured during the EAIIST traverse are available via PANGAEA at https://doi.pangaea.de/10.1594/PANGAEA.977763 (ref. ^34^). All samples were collected in full compliance with the Antarctic Treaty System and under the supervision of the French Polar Institute (IPEV), a COMNAP member, ensuring adherence to all legal and ethical requirements.

## Code availability

We used routines described in refs. ^27^ and ^60^ throughout this manuscript, which can be accessed at https://github.com/araudz/moist_isentropic_transport.
