# Supplementary material for: Water isotope–temperature relationship variability across Antarctica set by atmospheric circulation
Source: Nat Geosci. 2026 Apr 13;19(5):581–8. doi: 10.1038/s41561-026-01961-y (PMC13167469; doi:10.1038/s41561-026-01961-y)
Supplement: Supplementary file 1 — Supplementary Text 1–6, Figs. 1–8 and references 1–29. [file 41561_2026_1961_MOESM1_ESM.pdf]

nature geoscience

Supplementary information https://doi.org/10.1038/s41561-026-01961-y

Water isotope–temperature relationship variability across Antarctica set by atmospheric circulation

In the format provided by the authors and unedited

This PDF file includes:

Supplementary text S1 to S6
Figures S1 to S8
References (1 to 29)

S1. ***Focus on temporal variability in coastal regions***

From 18 November to 22 November, the EAIIST traverse was located at the site of D3, only 5.5 km away from the station DDU, which provides the opportunity for an in-depth comparison of the isotopic composition from two site in immediate proximity. Despite the close proximity, the sites of D3 and DDU are very different. DDU is located at 33 m a.s.l. on a rocky island surrounded by sea ice at the time of the traverse, but which can be open water in summer. In contrast, the D3 site is located at an altitude of 93 m a.s.l. on a blue ice slope 500m away from the coast on the actual continent.

During this period of five days, we observe at both sites two contrasting periods of diurnal variability. On 18–19 November, a weak diurnal cycle of temperature and humidity levels leads to almost no obvious isotopic variability, but the period 19–22 November has large diurnal cycles of temperature and wind speed that are associated with substantial humidity and water isotopic cycles (Fig. S1). Overall, temperature records show parallel diurnal cycles, with a significant offset of 1.1°C ($p < 0.005$ from a non-parametric one-way ANOVA test), partly due to the altitude difference (a dry adiabatic gradient of roughly -10°C/km would suggest a difference of 0.6°C between both sites). The mixing ratio show almost identical diurnal cycles, but for which the humidity levels are slightly higher at EAIIST (50 ppmv difference on average, $p = 0.02$), despite the temperature being lower. The difference is larger during day time period while the humidity levels at DDU and D3 converge toward the same value during the evenings when katabatic winds occur. For the water isotopes, we expect diurnal cycles [1], for which the origin is not directly associated with the temperature variations due to the change of insolation throughout the day. Coherent $\delta^{18}O$ cycles with no significant average difference ($p = 0.4$) are indeed observed during the period from 18 November to 22 November, despite the statistically significant temperature difference.

The measurements of the moisture isotopic composition at these two nearby locations provide key elements to evaluate the impact of the origin of the air masses on the isotopic composition in Polar coastal areas such as Adélie Land. Indeed, Figure S1 shows a significant impact of wind speed on the moisture isotopic composition, with periods of high wind speed (5 $m.s^{-1}$, red and blue shaded periods in Fig. S1) characterised by medium to low mixing ratio and lower $\delta^{18}O$ particularly during katabatic winds (down to -45‰, blue shaded in Fig. S1). Wind directions are unfortunately unavailable for these periods due to malfunction of the AWS sensors, but the katabatic identity is assigned based on known previous katabatic wind timing [1]. In order to explain the wide isotopic difference observed for the red (18/11 to 19/11) and blue (19/11 to 22/11) periods despite similar ranges of wind speed and temperature, we made use of air mass back-trajectories (see Section S2). As displayed in Figure 2.f), the period from 18 November to 19 November is characterised by a synoptic event during which moisture is transported to Dumont D'Urville along the coast of Victoria and Adélie Lands, whereas from 19 November to 22 November, air masses are advected toward Dumont d'Urville during intense katabatic winds starting around 12:00 UTC and ending around 23:00 UTC coming from the Plateau region.

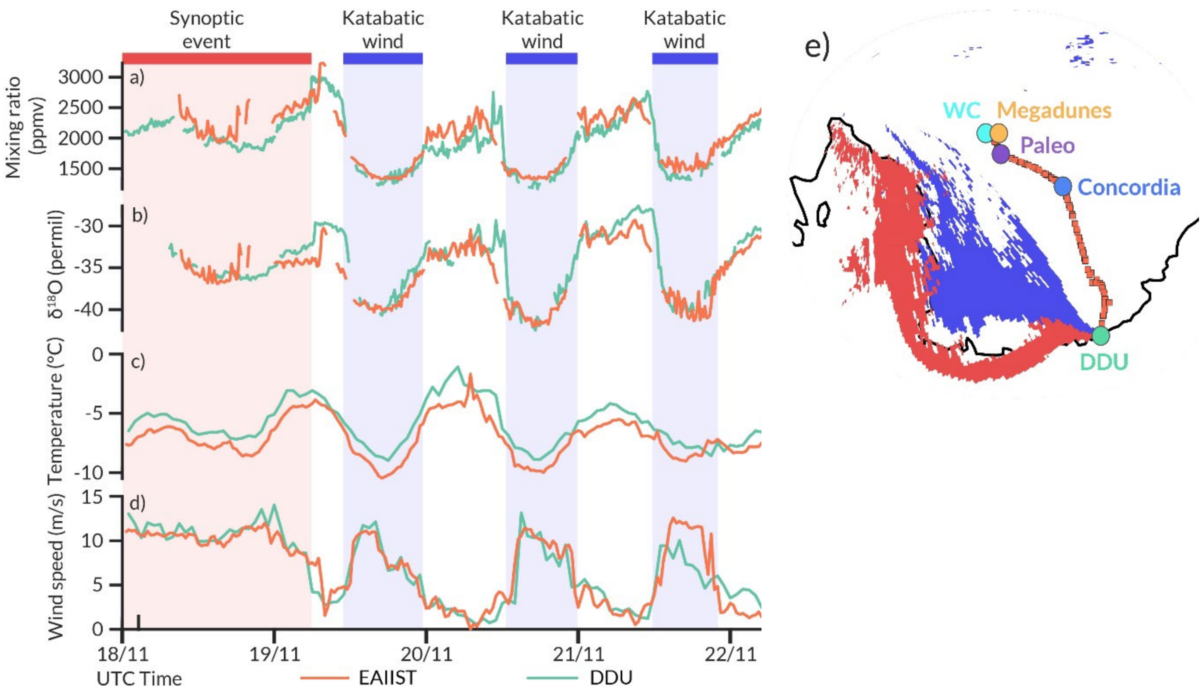

Figure S1: Focus on the isotopic composition diurnal variations in coastal areas: *Timeseries from 18 to 22 November 2019 of a) mixing ratios (ppmv), b) $\delta^{18}O$ (‰), c) temperature from weather stations (°C), d) wind speed (m/s) measured when the EAIIST traverse was at the D3 (orange line), in comparison with data from similar instruments at DDU, 5.5km away (green). The blue shaded areas indicate period with katabatic winds (>5 $m.s^{-1}$) when dry moisture from the continental ice sheet was advected toward DDU and D3, the red shaded area indicates the period during which a synoptic event brought moisture along the coast. e) clusters of back-trajectories during the period from 18/11 to 19/11, characterised by the synoptic event (red) and during the period from 19/11 to 22/11, characterised by katabatic winds (blue).*

S2. *Spatial and temporal variability of isotope and temperature at the synoptic scale*

From 1 December 2019 to 30 January 2020, the EAIIST traverse travelled across the East Antarctic Plateau. The meteorological conditions, and in particular the temperature, during this traverse were extremely similar to Dome C observations (normalised (1SD) RMS difference of temperatures below 0.8 over the entire period). Despite the similar temperature, we observed substantial differences in isotopic composition between the caravan and Dome C over the same period (normalised RMS difference of 3.2 for $\delta^{18}O$). Here, we delve into the difference between the vapour isotopic composition for different regions of the Plateau with similar climate.

When the traverse reached the plateau, it moved toward Concordia station and then continued further south toward the Megadunes area. Temperature and humidity observed on the traverse reached *Antarctic Plateau* conditions (Dome C-like conditions, with an average temperature of ~-35°C and 400ppmv) around 1 December 2019 until 30 January 2020. In contrast, the traverse's vapour $\delta^{18}O$ varied from -50 to -73‰ during this period, while Dome C vapour $\delta^{18}O$ remained bounded between -67 and -70‰, leading to significant differences of vapour $\delta^{18}O$ (up to 20‰) which persists except when the traverse reaches Dome C (4 December and 18 January).

Specifically, we focus on three case studies from stops of the traverse during which we have at least 12 hours of measurement. For each of these periods, the temperature at Dome C and the location of the EAIIST traverse are not significantly different (less than 1°C, $p > 0.05$ from a non-parametric one-way ANOVA test). For all of these periods, we obtain significant differences ($p < 0.05$) between the $\delta^{18}O$ at Dome C and on the EAIIST traverse, even when the distance is as low as 150km. Since the temperatures at both sites were very similar, the difference of water vapour isotopic composition can be linked with either different origin of the air masses, or different mixing in the atmospheric boundary layer.

The history of the air masses before arriving to the traverse or Concordia station was evaluated using the Flexpart Lagrangian model Lagrangian Particle Dispersion Model Flexpart 10 [2]. The model as forced using ERA5 hourly data and produced an ensemble of 10 000 back-trajectories with a three-hour timestep for 10 days. The ensemble of trajectories for these virtual particles were then clustered to evaluate the horizontal and vertical past history of the air masses. To do so, we used a threshold on the density of trajectories (defined as the calculation indicates the proportion of particles over the ten-days back-trajectory at each grid point relatively to total amount of particles) to evaluate the areas including the largest ensemble of trajectories.

We produced trajectories ending at the gridpoint of interest at 1000m altitude to be free from the local, small scale vertical mixing near the boundary layer. Yet, since our measurements were taken very much inside the atmospheric boundary layer (between 2 and 5 m above the ground), we also computed as well back-trajectories ending at 50m altitude. The difference, while noticeable, do not affect the qualitative interpretation of the results.

Using back trajectory analysis, we obtain a large range of contrasting vertical and horizontal origin of the air masses from similar horizontal history (Fig. S2a), to no horizontal overlap (Fig. S2e). For the first case, 01 December 2019, despite the proximity of the traverse with Dome C, similar climatic conditions, and similar horizontal air masses history, we observe significant difference in isotopic composition (Fig. S2a). In this case, the air masses were at different altitudes except for the last two days of the air masses history, and despite virtually equal final temperatures, they experience different temperatures for most of the history, and in particular when precipitation took place (6 days prior for the EAIIST traverse, against only 2 days prior for Dome C, Fig. S2b). In the two other cases, different origins with various amount of overlap explain the difference in final isotopic composition, despite again similar temperature. In all these cases, despite similar local conditions, the horizontal or vertical differences in air masses transport could lead to significant isotopic composition.

We also evaluated the stability of the atmospheric boundary layer by calculating the Richardson Number [3] in the boundary layer using the outputs of the regional model MAR, since it has been shown in Greenland that it can impact the vapour isotopic composition in the atmospheric boundary layer [4]. Overall, a much more variable Richardson number is observed at Dome C, with night-time values often larger than 0.25 (associated with a more stable boundary layer) and negative day-time values (associated with convection and strong mixing), than the other locations covered by EAIIST traverse on the Plateau where the Richardson number remains between 0 and 0.1. This suggests that the boundary layer at Dome C alternated between stable and stratified boundary layer and non-stable convective atmosphere [5], between night and day, respectively. These conditions, however, do not reflect those observed outside of the Dome which undergo a relatively strong mixing consistently during days and night as suggested by $R_i < 0.1$ [6]. During the six stops of the EAIIST traverse, the conditions at Dome C remained well mixed except during the Paleo stop on 17 December

2019. In this case, enhanced mixing between the boundary layer and the free atmosphere at Paleo could also explain the difference between the boundary layer isotopic composition.

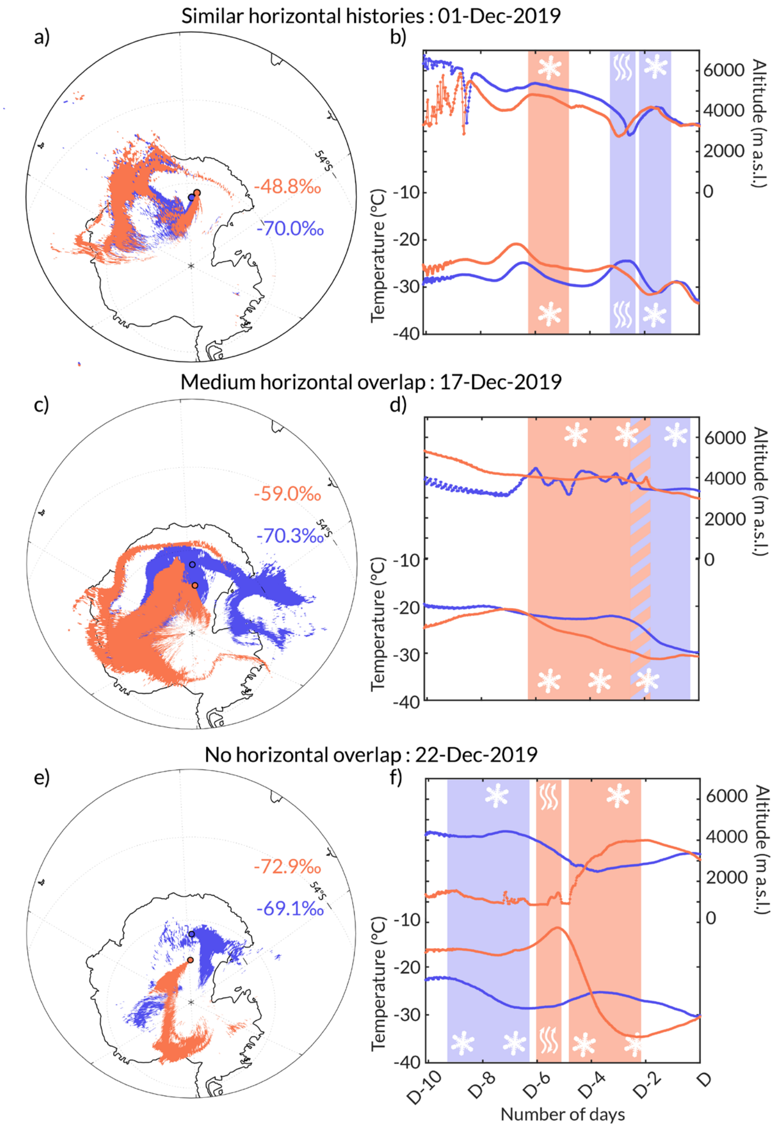

Figure S2: Back-trajectory analyses for stationary periods of the EAIIST traverse: *ensemble of back-trajectories reaching Dome C (Blue) and the location of the EAIIST traverse (orange) for three case studies: a) and b), 1 December, similar horizontal histories, c) and d) 17 December, medium horizontal overlap, and e) and f) no horizontal overlap. The average isotopic composition measured at each location is given ($\delta^{18}O$, orange and blue values on the map), as well as the altitude (m a.s.l.) and temperature (°C) as a function of time indicated in number of days before the event (b), d), f)). The shaded areas (orange for EAIIST, blue for Concordia, crossed for both) in panel b), d), and f) indicate either evidence of precipitation (snowflakes) or recycling (arrows) evaluated by decrease or increase of the mixing ratio, respectively.*

These differences in isotopic composition that exist despite similar climatic conditions reinforce that the link between isotopic composition and temperature is more complex than a simple linear regression, even for a place with relatively homogeneous conditions. Overall, the isotope-temperature relationship is calculated by averaging a large ensemble of synoptic conditions, either in the case of the average of several years of snow isotopic composition [7],

or long time series of precipitation isotopic composition [8]. Yet, this confirms that the link between local temperature and isotopic composition is actually not direct, but comes from the link of both these variables with the temperature gradient along the distillation pathway. As a result, this suggests that we can use the vapour measurements presented in this manuscript as an indicator of large scale Rayleigh distillation pattern at the day-to-day scale, rather than as indicator of only local exchange within the atmospheric boundary layer for which only the local temperature and snow isotopic composition would matter.

S3. Comparison between snow and vapour isotopic composition

To further evaluate whether the vapour isotopic composition variations can be explained by the local exchange with surface snow, we compare at Dome C and across the EAIIST traverse the vapour and snow isotopic composition (Figure S3). There is ample evidence that the vapour isotopic composition diurnal cycle is dominated by sublimation and condensation near the surface with moisture fluxes restricted to the atmospheric boundary layer [9–14]. Here, at the day-to-day scale, we evaluate the impact of variations of snow isotopic composition on the vapour isotopic composition (Fig. S3a). Over the entire traverse, we observe a relatively good correlation linked with the parallel Rayleigh distillation from the coast to Dome C. On the plateau over the traverse and at Dome C, the correlation is completely destroyed ($r^2 = 0.03$, $p > 0.5$ for EAIIST for snow $\delta^{18}O$ < 45‰). This confirms that at the day-to-day scale or spatially over the Antarctic Plateau, the vapour isotopic composition is not controlled directly by the snow isotopic composition.

We evaluated if the isotopic fractionation during sublimation or condensation could be responsible for the difference between the snow and vapour isotopic composition variations on the East Antarctic Plateau by computing the impact of fractionation at the average temperature of the day. We do not find a link between the vapour isotopic composition and the vapour that would be at equilibrium with the snow (Fig. 3b), and the spatial correlation over the Plateau or the temporal correlation at Dome C are not improved with p values remaining above 0.5. We did not take into account kinetic fractionation which are mainly associated with second order impacts on the isotopic composition.

This additional line of evidence suggests that the vapour isotopic composition during the EAIIST traverse and at Dome C is not controlled by the local exchanges with the snow at the day-to-day scale.

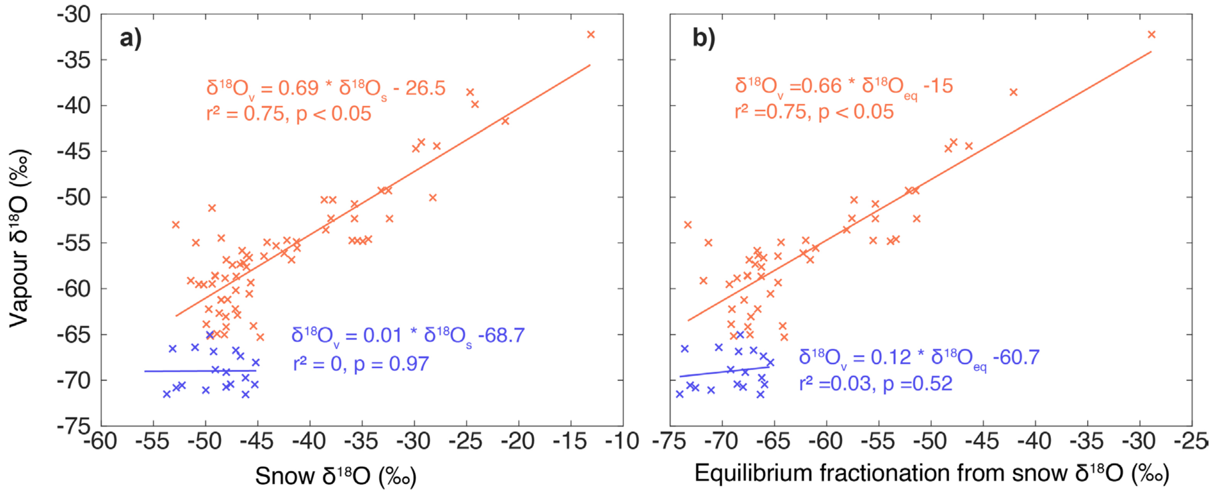

Figure S3: Comparison of vapour and surface snow isotopic composition at the day-to-day scale: *a) vapour and surface snow isotopic composition on EAIIST (orange) and at Dome C (blue) during the traverse, b) vapour and calculation of vapour isotopic composition at equilibrium with the surface snow taking into account the average temperature of each day on EAIIST (orange) and at Dome C (blue). p-values were obtained from one-sided statistical analysis.*

S4. Evaluation of the spatial variability of the temporal and spatial isotope relationships in the model outputs

[15] showed a strong variability in the isotope-temperature relationship for even nearby gridpoints of GCM outputs in Antarctica. We found similar results here which justify that only qualitative comparison can be made from the model outputs. Figure S4 presents the ensemble of slopes found for all of Antarctica for the three models we studied here. For all three models, we show that the spatial isotope temperature relationship is larger overall than the temporal ones, at both the seasonal and interannual scales. Indeed, spatial variability is characterised by slopes around or larger than 0.8‰.°C^{-1}, in agreement with observations from surface snow isotopic composition gathered at local (less than 100km) [16,17] or global scale [18,19]. In contrast, temporal slopes at the seasonal scales are range between 0.2 and 0.8‰.°C^{-1}, with a very good agreement between the temporal patterns obtained in models (in particular ECHAM6-wiso and LMDZ-iso) and observations (Fig. S4b) [16,17,20–25]. At the interannual scales, temporal slopes are more variable, largely because of the small amount of datapoints (45 years), and the small interannual variations of temperature (ΔT) which are affecting a lot the linear regression. The values associated with a significant relationship range between 0.5 and 1‰.°C^{-1}, and the outputs for all the models are in agreement with the single datapoints of interannual isotope-temperature that is found in the literature [22]. Despite the very different spatial seasonal relationships simulated by all three models, with only five sites with observations, it is difficult to evaluate directly in which model to put the most trust.

Overall, the physics of isotope-enabled GCM seems like the most robust approach to obtain the isotope-temperature relationship that should be applied to an ice core record. While there are still some limitations from the current version of the parametrisations, the latest CMIP6 versions show that they are able to produce accurate isotope-temperature relationships which can be used to study past climate variability from ice core records. It is for instance interesting to note that the spatial patterns of isotope-temperature relationships are the same for LMDZiso and ECHAMwiso despite the difference in the physics of the model, which support a larger trust in the spatial patterns which mostly reproduce the spatial patterns obtained from the very patchy range of observations. It is worth noting that both models are nudged against ERA5 reanalysis, but ECHAM is nudged using 3D ERA5 fields of vorticity and divergence (comparable to nudging of horizontal winds) and also to 3D ERA5 temperature data while LMDZiso is nudged directly to horizontal winds and temperature fields.

Instead, we suggest that using the isentropic framework to evaluate which part of the underlying climatic conditions are changing is the key to validate the outputs of the isoGCMs. Indeed, this physically based validation provides a framework to understand what is the leading cause of the temporal isotope-temperature relationship, and can be a validation of the of the model predictions. Considering the spatial patterns, and the low confidence in the interannual slopes due to the small number of years considered, the seasonal isotope-temperature relationship predicted by the iso-GCM for the interpretation of water isotopes in ice core records is so far the best estimate at time scales shorter than decadal. In particular, the good agreement between the ECHAM6-wiso and LMDZ6iso outputs and the observations suggest it is a really tool to generalise the isotope-temperature relationships to every grid point of

Antarctica, for instance, when providing reconstructions of the last 2000 years [8,26]. Generalisation to past periods with potentially different underlying climate states should be done on a case- by-case basis, evaluating how realistic is the change of the atmospheric circulation. For instance, we do not expect the underlying climate conditions to remain the same in glacial periods where the ice sheet thickness and the sea ice extent are extremely different [27].

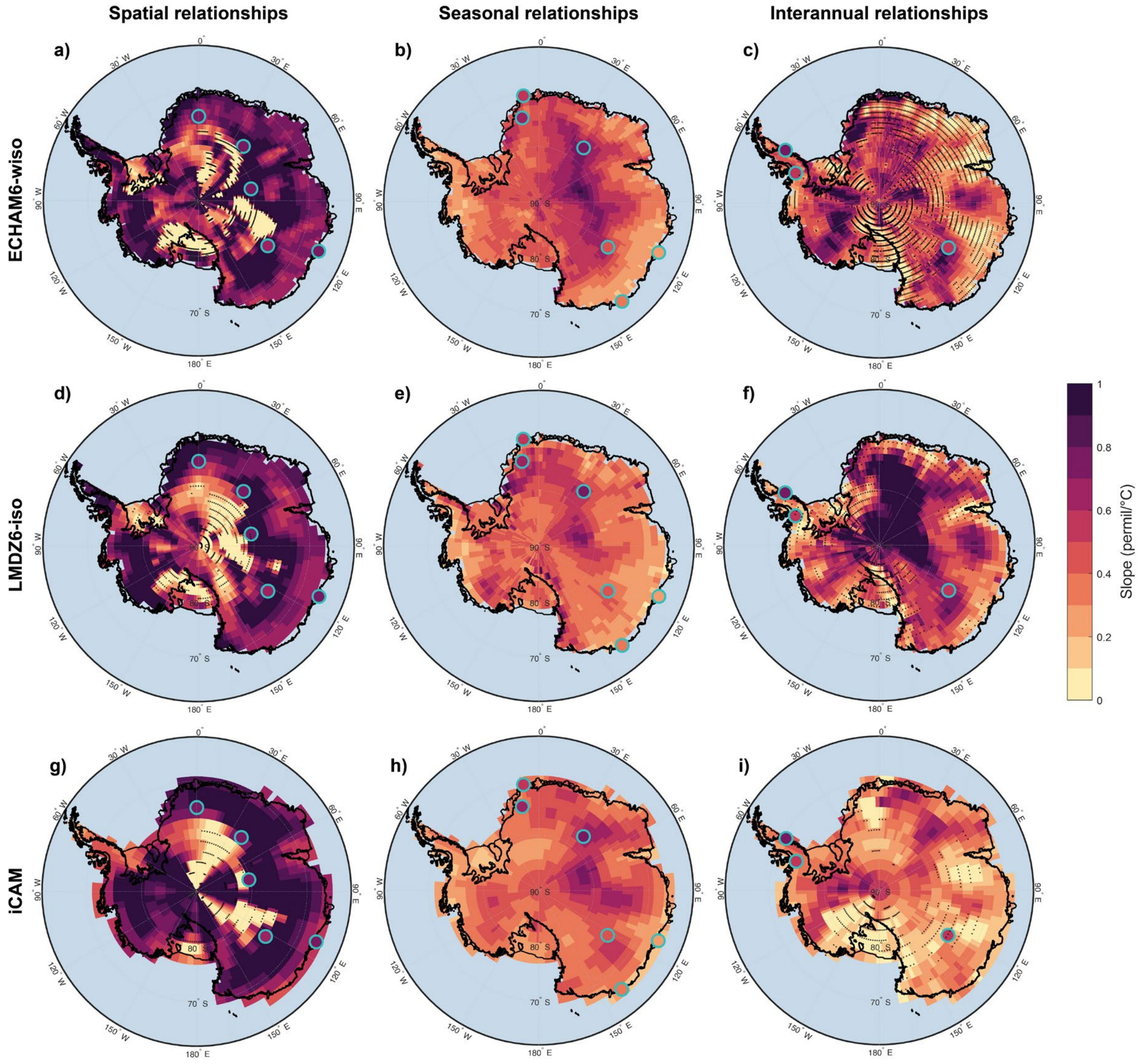

Figure S4: Variability of the isotope-temperature relationships across Antarctica: *isotope-temperature slope in the precipitation model outputs from ECHAM6-wiso (a, b, c), LMDZ6-iso (d,e,f), and CESM2-iso (g,h,i) computed spatially across the neighbouring gridpoints (a, d, and g, taking into account the square gridpoints in latitude and longitude, total 25 points), temporally focusing on the seasonal relationship (b,e,h) or on the interannual relationship (c,f,i). Slopes reported in the literature for given locations are indicated with circles with a teal contour. Datapoints with non significant linear relationships (one sided, p > 0.05) are indicated with black dots.*

S5. Impact of pollution from engines' combustion

The impact of potential pollution from the water emitted by the combustion of fuels by the engine of the vehicles used on the traverse and the power generator has been evaluated, and datapoints for which suspicion of contamination from the engine moisture were flagged (highlighted with a lighter orange in Fig. 1). This potential contamination was identified by elevated water mixing ratio compared to the outputs of the regional model MAR for the traverse's location, as well as when spikes of methane were detected by the Picarro (as previously done in Casado et al, 2016). This was particularly the case when the EAIIST traverse stopped at Dome C where the caravan was in an area at the centre of the station (see Figure S5) with a lot of activity, and in particular tractors continuously operating. It was also notably the case during the megadune period (Fig. 1). During these periods, the $\delta^{18}O$ was significantly elevated, with difference larger than the accuracy (see Methods).

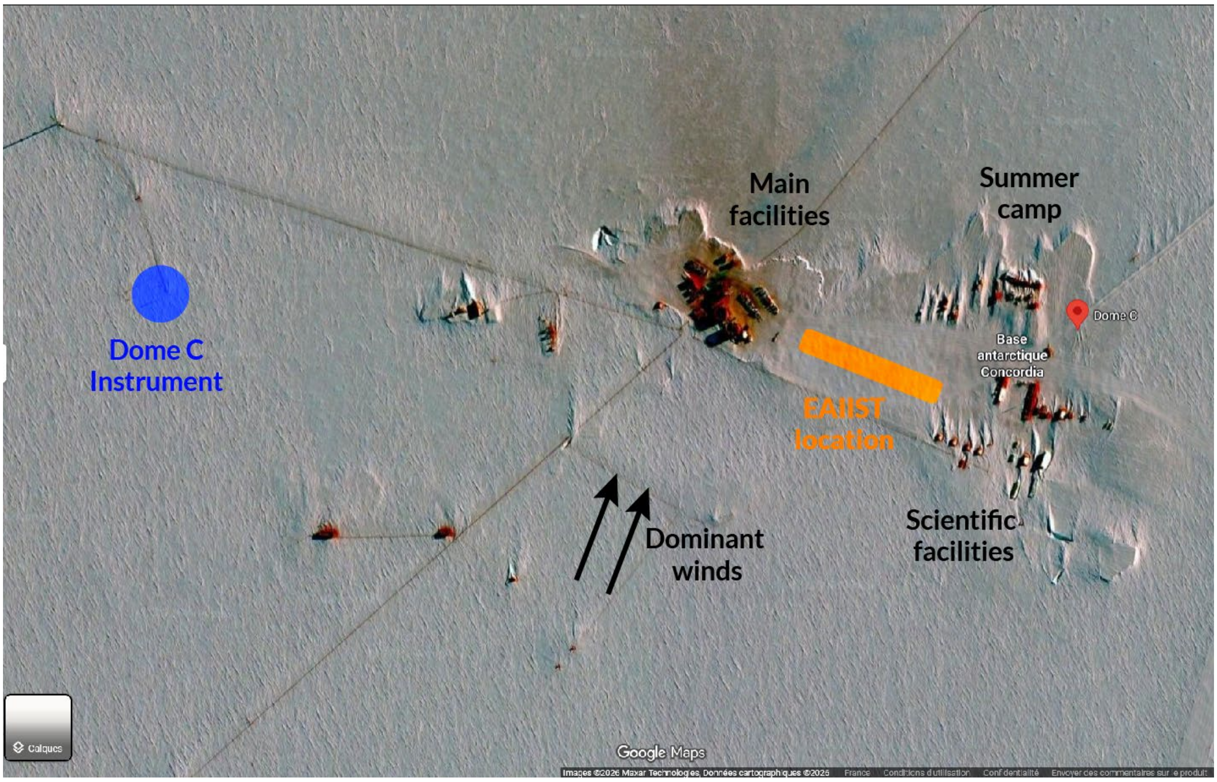

Figure S5: satellite view of the Dome C station: *location of the EAIIST traverse during the stop at Dome C in the centre of the station compared to the Dome C fixed instrument at the edge of the clean area. Screenshot realised from Google Maps over Concordia Station.*

The impact of pollution is clearly visible on the snow (Figure S5), and we suspect that exhausts could also influence the isotopic composition measured on the EAIIST traverse in certain conditions. Indeed, the water produced during combustion of petrol by engines has a peculiar isotopic composition characterised by $\delta^{18}O$ values between +5 and +25‰ and δD values around -150‰ [28,29]. As discussed in Section S2, at Dome C, conditions with relatively large Richardson number are conducive to inversion layers and stratified boundary layers, during which moisture from the exhaust could accumulate in the atmospheric boundary layer.

In such conditions, we evaluated that the impact of an arbitrary value of 10% of the total moisture originating from combustion of petrol could lead to a bias on the $\delta^{18}O$ between +7.5 and +9‰, and on the dexcess between -24 and -28‰. Due to logistical hindrance, there are few days during which we obtained concomitant measurement from the EAIIST traverse and the Dome C instrument, even when the traverse was at Dome C. We have common measurements on 7 December 2019 as well as on 23 January 2020. The difference between

the $\delta^{18}O$ on these two days are significantly larger than the expected accuracy with roughly 2 and 8‰, for 7 December and 23 January, respectively (Fig. S6). There are also large differences for the dexcess, in particular with more than -15‰ difference on 23 January, but due to the poor accuracy, it is difficult to assess if these are significant. We suggest that the logistical operations on the EAIIST traverse at the site of Dome C could have led, due to moisture generated by the exhausts, to the anomalous signal. Indeed, relatively small amount of moisture originating from combustion could lead to significant impact on the atmospheric $\delta^{18}O$. Outside of the site of Dome C, we only observe similar contamination from exhaust moisture during the stop at Megadunes. These sites were the ones with the lowest windspeed, and the largest Richardson numbers of the traverse, which could suggest that the conditions were conducive for the atmospheric boundary layer to be filled by exhaust moisture. No evidence of contamination (no elevated moisture content as well as no CH4 spikes) has been observed for the rest of the traverse, eliminating suspicions that such artefact increase of $\delta^{18}O$ by exhaust moisture occurred.

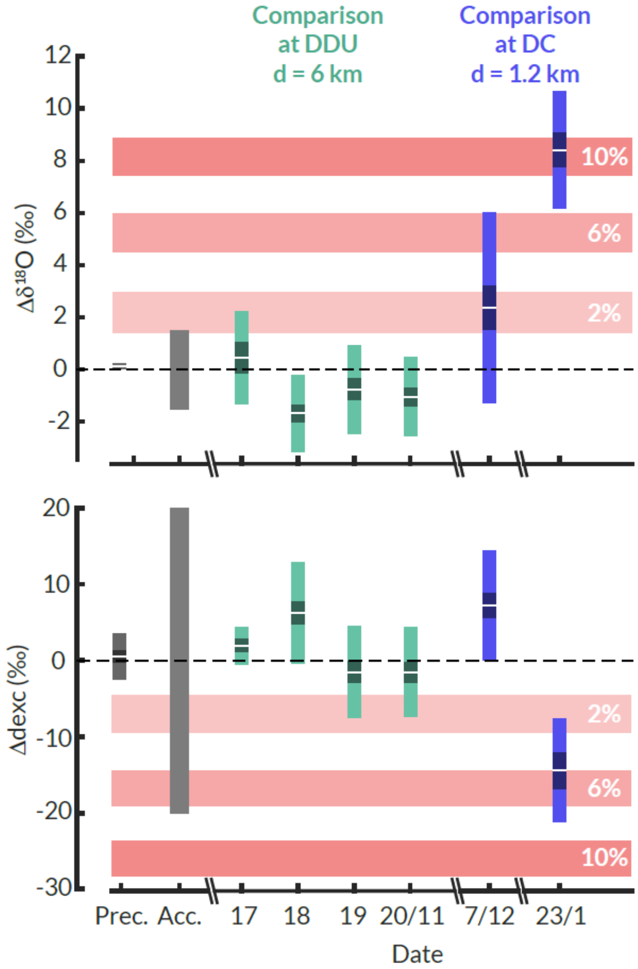

Figure S6: Comparison of isotopic compositions measured at DDU (green) and Dome C (blue) when two instruments were available: *evaluation of the difference between the $\delta^{18}O$ (top) and the dexcess (bottom) measured on the EAIIST traverse and by the fixed instrument at Dumont d'Urville station (DDU) and Dome C station (DC). The impact of a given amount of moisture originating from engine exhausts (in percent of the total amount) on the isotopic composition is indicating in different shades of red, in comparison with the precision (Prec.) and accuracy (Acc.) estimated in the methods (grey bars).*

S6. ***Water Vapour Isotopic Composition Measurements***

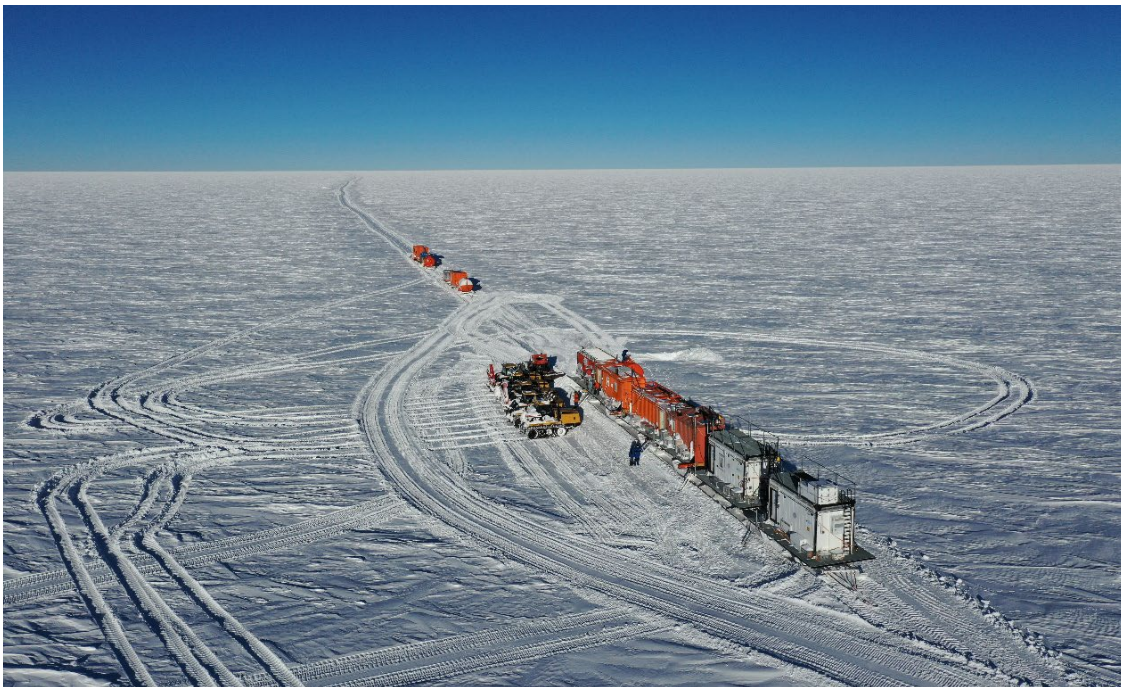

a)

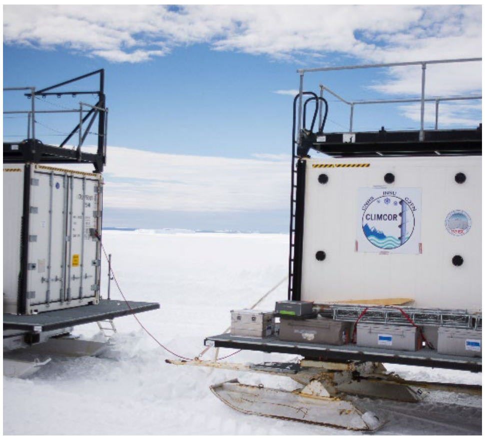

b)

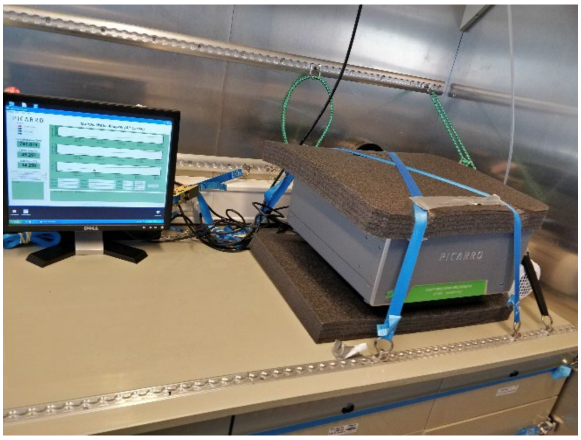

c)

Figure S7: *(a) Drone photo of the convoy during a night stop on the East Antarctic Plateau; (b) the Picarro prior to installation inside the warm lab; (c) the Picarro analyser secured to the lab desk.*

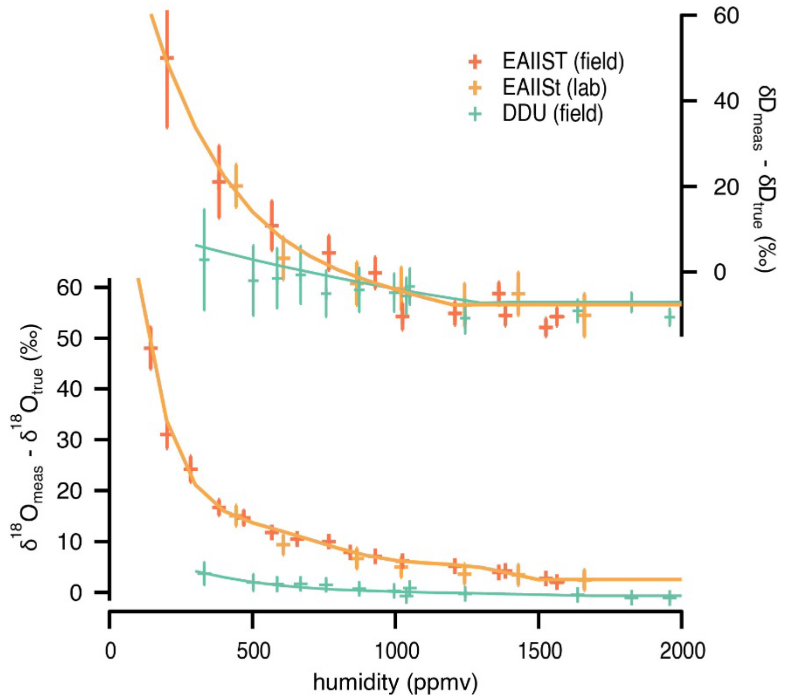

Figure S8: *Humidity dependence calibration for the EAIIST instrument in the field (DDU, dark orange) and laboratory (LSCE, light orange) compared to the DDU station's permanent instrument. Vertical bars show two standard deviations from the mean for each calibration (calculated from measurements over 30 minutes at roughly 1 Hz, i.e. N = 1800).*

Bibliography

1. Bréant, C. *et al.* Coastal water vapor isotopic composition driven by katabatic wind variability in summer at Dumont d'Urville, coastal East Antarctica. *Earth Planet. Sci. Lett.* **514**, 37–47 (2019).

2. Pisso, I. *et al.* The Lagrangian particle dispersion model FLEXPART version 10.4. *Geosci. Model Dev.* **12**, 4955–4997 (2019).

3. Richardson, L. F. *The Supply of Energy from and to Atmospheric Eddies*. vol. 97 (1920).

4. Berkelhammer, M. *et al.* Surface-atmosphere decoupling limits accumulation at Summit, Greenland. *Sci. Adv.* **2**, (2016).

5. King, J. C., Argentini, S. A. & Anderson, P. S. Contrasts between the summertime surface energy balance and boundary layer structure at Dome C and Halley stations, Antarctica. *J. Geophys. Res. Atmos.* **111**, n/a--n/a (2006).

6. Zilitinkevich, S. S. *et al.* Turbulence energetics in stably stratified geophysical flows: Strong and weak mixing regimes. *Q. J. R. Meteorol. Soc.* **134**, 793–799 (2008).

7. Lorius, C., Merlivat, L. & Hagemann, R. Variation in the mean deuterium content of precipitations in Antarctica. *J. Geophys. Res.* **74**, 7027–7031 (1969).

8. Stenni, B. *et al.* Antarctic climate variability on regional and continental scales over the last 2000 years. *Clim. Past* **13**, 1609–1634 (2017).

9. Casado, M. *et al.* Continuous measurements of isotopic composition of water vapour on the East Antarctic Plateau. *Atmos. Chem. Phys.* **16**, 8521–8538 (2016).

10. Casado, M. *et al.* Archival processes of the water stable isotope signal in East Antarctic ice cores. *Cryosph.* **12**, 1745–1766 (2018).

11. Ritter, F. *et al.* Isotopic exchange on the diurnal scale between near-surface snow and lower atmospheric water vapor at Kohnen station, East Antarctica. *Cryosph.* **2016**, 1–35 (2016).

12. Vignon, E. *et al.* Stable boundary layer regimes at Dome C, Antarctica: observation and analysis. *Q. J. R. Meteorol. Soc.* (2017).

13. Wahl, S., Steen-Larsen, H. C. & Reuder, J. Quantifying the Stable Water Isotopologue Exchange between Snow Surface and Lower Atmosphere by Direct Flux Measurements. *J. Geophys. Res. Atmos.* e2020JD034400 (2021).

14. Wahl, S. *et al.* Atmosphere-Snow Exchange Explains Surface Snow Isotope Variability. *Geophys. Res. Lett.* e2022GL099529 (2022).

15. Goursaud, S., Masson-Delmotte, V., Favier, V., Orsi, A. & Werner, M. Water stable isotope spatio-temporal variability in Antarctica in 1960--2013: observations and simulations from the ECHAM5-wiso atmospheric general circulation model. *Clim. Past* **14**, 923–946 (2018).

16. Touzeau, A. *et al.* Acquisition of isotopic composition for surface snow in East Antarctica and the links to climatic parameters. *Cryosph.* **10**, 837–852 (2016).

17. McMorrow, A. J. *et al.* Intercomparison of firn core and meteorological data. *Antarct. Sci.* **13**, 329–337 (2001).

18. Masson-Delmotte, V. *et al.* A Review of Antarctic Surface Snow Isotopic Composition: Observations, Atmospheric Circulation, and Isotopic Modeling*. *J. Clim.* **21**, 3359–

3387 (2008).

19. Landais, A. *et al.* Surface studies of water isotopes in Antarctica for quantitative interpretation of deep ice core data. *Comptes Rendus Geosci.* (2017).

20. Landais, A., Ekaykin, A., Barkan, E., Winkler, R. & Luz, B. Seasonal variations of 17O-excess and d-excess in snow precipitation at Vostok station, East Antarctica. *J. Glaciol.* **58**, 725–733 (2012).

21. Stenni, B. *et al.* Three-year monitoring of stable isotopes of precipitation at Concordia Station, East Antarctica. *Cryosph.* **2016**, 1–30 (2016).

22. Dreossi, G. *et al.* A decade (2008–2017) of water stable isotope composition of precipitation at Concordia Station, East Antarctica. *Cryosph.* **18**, 3911–3931 (2024).

23. Fujita, K. & Abe, O. Stable isotopes in daily precipitation at Dome Fuji, East Antarctica. *Geophys. Res. Lett.* **33**, (2006).

24. Schlosser, E., Reijmer, C., Oerter, H. & Graf, W. The influence of precipitation origin on the d18O-T relationship at Neumayer station, Ekströmisen, Antarctica. *Ann. Glaciol.* **39**, 41–48 (2004).

25. Leroy-Dos Santos, C. *et al.* From atmospheric water isotopes measurement to firn core interpretation in Adélie Land: a case study for isotope-enabled atmospheric models in Antarctica. *Cryosph.* **17**, 5241–5254 (2023).

26. Casado, M., Hébert, R., Faranda, D. & Landais, A. The quandary of detecting the signature of climate change in Antarctica. *Nat. Clim. Chang.* (2023).

27. Holloway, M. D. *et al.* Antarctic last interglacial isotope peak in response to sea ice retreat not ice-sheet collapse. *Nat. Commun.* **7**, 12293 (2016).

28. Gorski, G. *et al.* Vapor hydrogen and oxygen isotopes reflect water of combustion in the urban atmosphere. *Proc. Natl. Acad. Sci.* **112**, 3247–3252 (2015).

29. Fiorella, R. P., Bares, R., Lin, J. C., Ehleringer, J. R. & Bowen, G. J. Detection and variability of combustion-derived vapor in an urban basin. *Atmos. Chem. Phys.* **18**, 8529–8547 (2018).
